# A molecular survey of spotted fever group rickettsiae in introduced raccoons (*Procyon lotor*)

**DOI:** 10.1186/s13071-022-05280-0

**Published:** 2022-05-07

**Authors:** Joanna Hildebrand, Agnieszka Perec-Matysiak, Marcin Popiołek, Dorota Merta, Izabella Myśliwy, Katarzyna Buńkowska-Gawlik

**Affiliations:** 1 Department of Parasitology, University of Wrocław, Wrocław, Poland; 2 Department of Ecology and Environmental Protection, Institute of Biology, Pedagogical University of Kraków, Kraków, Poland

**Keywords:** *Rickettsia*, Spotted fever group rickettsiae, Raccoons

## Abstract

**Background:**

The raccoon *Procyon lotor* (Linnaeus, 1758) (Carnivora; Procyonidae) is one of the most important and most intensively studied invasive mammal species in Europe. Within the last 30 years the raccoon has spread at an increasing rate, resulting in the establishment of local populations in various regions of Europe. In these newly colonised areas, gaps in knowledge of the raccoon’s biology concern not only most aspects of its ecology in a broad sense, but also its pathogens and parasites. Most micropathogens recorded hitherto in the raccoons that have colonised Europe have documented epizootic and zoonotic potential. Thus, it is considered especially important to investigate the role played by the raccoon in the spread of pathogens through both animal-animal and animal-human pathways.

**Methods:**

Tissue samples of raccoons from Poland and Germany were examined in this study. In total, 384 tissue samples from 220 raccoons (170 spleen samples, 82 liver biopsies, 132 ear biopsies) were examined using molecular methods. The presence of *Rickettsia* spp. DNA was screened through amplification of a fragment of the *gltA* gene. Samples that were PCR positive for *gltA* were tested for other rickettsial genes, *ompB* and a 17-kDa antigen. For taxonomic purposes, the obtained sequences were compared with corresponding sequences deposited in GenBank using the Basic Local Alignment Search Tool, and phylogenetic analyses were conducted using Bayesian inference implemented in MrBayes software.

**Results:**

*Rickettsia* DNA was confirmed only in skin biopsies; no isolates from the spleen or liver were positive for *Rickettsia* DNA. With the exception of one sample from Germany, which was positive for *Rickettsia helvetica* DNA, all the samples positive for *Rickettsia* DNA derived from the Polish population of raccoons. DNA of *Rickettsia* spp. was detected in 25 samples, i.e. 11.4% of the tested raccoons, and *R. helvetica* was confirmed in 52% of the positive samples. Additionally, single cases of *Rickettsia monacensis*, *Rickettsia raoultii*, and *Candidatus* Rickettsia kotlanii-like were found, and in 32% of all the positive samples similarity was shown to different *Rickettsia* endosymbionts. Out of the samples that tested positive for *gltA*, amplicons of *ompB* and 17 kDa were successfully sequenced from 14 and three samples, respectively.

**Conclusions:**

To the best of our knowledge, this study provides, for the first time, evidence of the occurrence of *Rickettsia* pathogens and endosymbionts in the European population of raccoons. Further, broader research on different species of wild vertebrates, and ticks, as potential vectors and hosts for tick-borne pathogens, in natural as well as in peri-urban environments, is therefore required.

**Graphical abstract:**

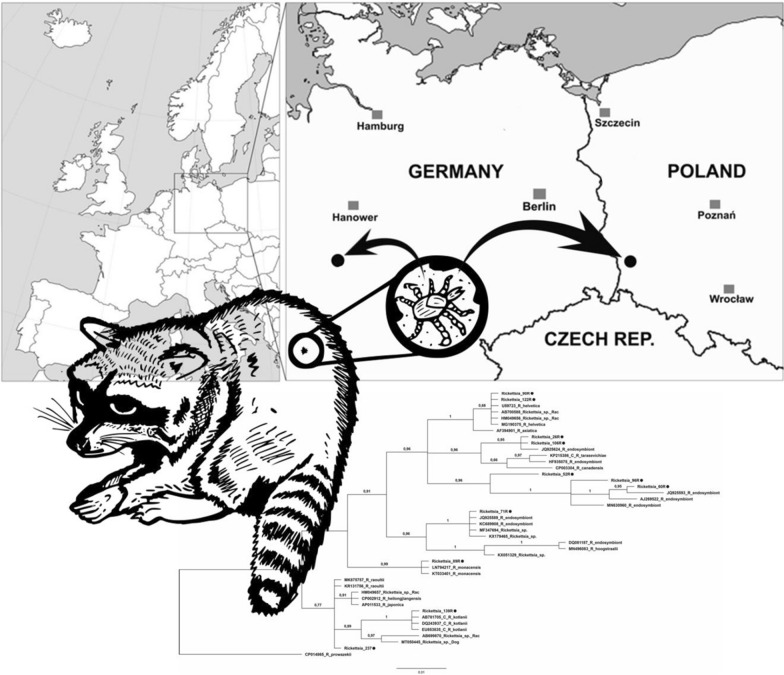

## Background

The raccoon *Procyon lotor* (Linnaeus, 1758) (Carnivora; Procyonidae) is one of the most important and most intensively studied invasive mammal species in Europe. Its natural distribution includes much of North America, from southern Canada to Panama. It was first introduced into Europe in the early twentieth century to meet the demands of the then rapidly developing fur industry. Local raccoon populations comprise escapees from fur farms and individuals that have been intentionally released into the wild. The geographical distribution of the species in Europe underwent widespread expansion ca. 20 years after its introduction, mainly from Germany and—to a lesser extent—from Belarus [1, 2]. During the last 30 years the raccoon has spread at an increasing rate, resulting in the establishment of local populations in various regions of Europe. In these newly colonised areas, gaps in knowledge of the raccoon’s biology concern not only most aspects of its ecology in a broad sense, but also its pathogens and parasites. While knowledge of parasitic helminths in European raccoon populations is slowly but steadily improving [3–14], knowledge of the parasitic protozoans and bacterial pathogens of this host is still insufficient and fragmentary [15–17]. The possibility that the raccoon may be able to transmit pathogens that are new to the Old Continent’s fauna, together with its phenomenal habitat plasticity, omnivorous diet, opportunism, synanthropy and synurbization, suggest that it may pose a risk as a new reservoir of pathogens for European mammals. Though the species composition of the parasites of the European raccoon populations is still being studied, the results of earlier studies indicate that the problem of introduced parasites pertains mostly to parasitic helminths, e.g. one of the most important zoonotic parasites of raccoons is the nematode *Baylisascaris procyonis*. The species compositions of parasitic protozoans and bacterial pathogens of this carnivore in North America and Europe differ somewhat. Leśniańska et al. [15] and Hildebrand et al. [16] showed that the microparasites and micropathogens recorded in the European raccoon populations are not the same as those recorded in North American populations. On the one hand, this suggests that an obstacle to the transcontinental transmission of these pathogens may be, for example, a lack of adequate vectors of these organisms in the environments newly colonised by the raccoons. On the other hand, raccoons colonising new areas of Europe may acquire new pathogens, and thus may play a role as a potential new environmental reservoir for them. Most micropathogens recorded hitherto in the raccoon colonists of Europe have documented epizoonotic and zoonotic potential. Raccoons co-occurring with native carnivores are increasingly encountered near human habitations. Thus it seems especially important to examine the part played by this species in the spread of pathogens through both animal-animal and animal-human pathways.

Rickettsiae are strictly intracellular vector-borne bacteria which are transmitted to vertebrates by a variety of arthropods, primarily fleas and ticks. The genus *Rickettsia* includes over 20 validated species, which have been classified into five groups: the spotted fever group (SFG) including *Rickettsia helvetica*; the transitional group including *Rickettsia felis*; the typhus group; the ancestral group *Rickettsia bellii* and *Rickettsia canadensis* [18, 19]; and an ever-growing number of unnamed and non-cultivated genotypes [20]. Some of these newly identified rickettsiae have been proven to be the causative agents of emerging human diseases; they are often first recognized through their associations with different animals and their ectoparasites and only later detected in clinical specimens and associated with specific diseases [21]. To date, 18 species of * Rickettsia* are recognized human pathogens [22]. In Europe, rickettsioses are well documented, and there are several circulating *Rickettsia* species and *Candidatus* Rickettsia species [23].

Molecular studies on the occurrence of *Rickettsia* spp. in populations of wild carnivores are scarce in comparison to the large number of studies that have been undertaken on *Rickettsia* spp. in vectors. The occurrence of *Rickettsia* spp. among raccoons has been reported in Hokkaido, Japan from a molecular study. To the best of our knowledge, no *Rickettsia* strain has been isolated from raccoons in Europe so far. Therefore, little is known about the role of raccoons in the epidemiological cycles of *Rickettsia* in Europe, or elsewhere in the world. Thus, the aim of the present study was to investigate, through polymerase chain reaction assays, the presence of potentially zoonotic agents, such as *Rickettsia* spp., in the tissues of invasive raccoons (*P. lotor*) in Poland and Germany. This survey is a first step in achieving a full understanding of the host–parasite relationship between invasive raccoons and *Rickettsia* spp. Knowledge of the genetic diversity of* Rickettsia* species and their reservoirs is useful for predicting the potential risk of infection posed by them and for making decisions regarding their effective management. The isolation of new *Rickettsia* from wild carnivores such as raccoons will contribute to an understanding of the complexity of these bacteria in wildlife.

## Methods

### Sample collection

Raccoons were sampled from Poland (Ruszów Forest District, Zgorzelecka Forest) and Germany (districts of Kassel and Dresden). Ruszów Forest District is located in the western part of the Lower Silesian Wilderness—the largest lowland forest complex in Europe. It is part of the large, compact forest complex of the Bory Dolnośląskie Forest, which has a low proportion of deciduous forest types and a high proportion of coniferous forest types [24]. We used tissues in the present study that were obtained through collaboration with other projects financed by different grants. The raccoon carcasses comprised those obtained from hunters undertaking raccoon culling as part of game management activities, road kills, and those collected during a predator control operation conducted as a part of a program to reintroduce the capercaillie (*Tetrao urogallus*) into the Lower Silesian Forest, which was co-financed by the European Commission. Ear, spleen and liver samples were obtained, if possible, during the autopsies, and stored at − 20 °C until analysis. In total, 384 tissue samples (170 spleen samples, 82 liver biopsies and 132 ear biopsies) from 220 raccoons were examined (Table 1).

**Table 1:** Number of sampled raccoons with regard to the type of tissue examined

Country	No. of individuals examined for specific tissues			
	Ear biopsies	Spleen samples	Liver biopsies	Total
Poland	50	73	56	179
Germany	0	15	26	41
Total	50	88	82	220

### Molecular analysis

DNA was extracted using the Bio-Trace DNA Purification Kit (EURx, Poland) in accordance with the manufacturer’s instructions, and stored at − 20 °C until further use. DNA concentrations were determined with a NanoDrop 2000 spectrophotometer (Nanodrop Technologies, Wilmington, DE).

The presence of *Rickettsia* spp. DNA was determined through the amplification of a 338-bp fragment of the *gltA* gene, which has conserved regions shared by all known *Rickettsia* species, in nested PCR using two primer sets, RpCS.877p-RpCS.1258n and RpCS.896p-RpCS.1233n [25]. All the *gltA*-positive samples were further examined using nested PCR assays amplifying parts of the other protein coding genes examined, namely *ompB* (primers Rc.rompB.4362p, Rc.rompB.4,836n, Rc.rompB.4,496p, Rc.rompB.4,762n) [26] and a 17-kDa antigen (primers 17 k-5, 17 k-3, 17KD1, 17KD2) [27]; the resulting 355-bp and 434-bp products were considered to indicate positive samples. Each PCR reaction was performed with 2× PCR Mix Plus (A&A Biotechnology, Gdynia, Poland) in a total reaction volume of 25 μl containing 1 μl of each primer (10 μM) and 3 μl (first reaction) or 1 μl (second reaction) of the DNA sample. Negative controls with nuclease-free distilled water, in the absence of template DNA, were included for each PCR reaction. The PCR products were subjected to electrophoresis on a 1.5% agarose gel, and stained with Midori Green stain (Nippon Genetics). In order to prevent contamination of the PCR, DNA extraction, reaction setup, PCR amplification and electrophoresis were performed in separate rooms.

The selected amplicons were purified using Exo-BAP (EURx) and directly sequenced in both directions by Macrogen (Amsterdam, the Netherlands) with the primers used for DNA amplification. Finally, the nucleotide sequences obtained in this study were edited using DNA Baser Sequence Assembly software (Heracle BioSoft, Romania) and compared with each other and with corresponding sequences registered in GenBank using the National Center for Biotechnology Information (NCBI) Basic Local Alignment Search Tool (BLAST) program [http://blast.ncbi.nlm.nih.gov/Blast.cgi].

Phylogenetic analyses were conducted using Bayesian interface implemented in MrBayes v3.2.7 software [28]. The general time-reversible model with estimates of invariant sites and gamma-distributed among-site variation (GTR + G + I) model was identified as the best-fitting nucleotide substitution model for both *gltA* and *ompB* alignments using MEGA X software [29]. The generated consensus trees were visualized using FigTree ver. 1.4.4 software [30].

## Results

A total of 220 raccoons collected from two localities in Germany and two localities in Poland were tested for the presence of rickettsial DNA. Due to limited access to a complete range of tissues from all the specimens from each location, only one type of tissue was examined for some of the individual raccoons (Table 1). However, due to a lack of data on the role of raccoons in *Rickettsia* sp. circulation in Europe, all the results are presented here. In total, 384 tissue isolates derived from the skin, spleen and liver were used in this study.

**Fig. 1:**
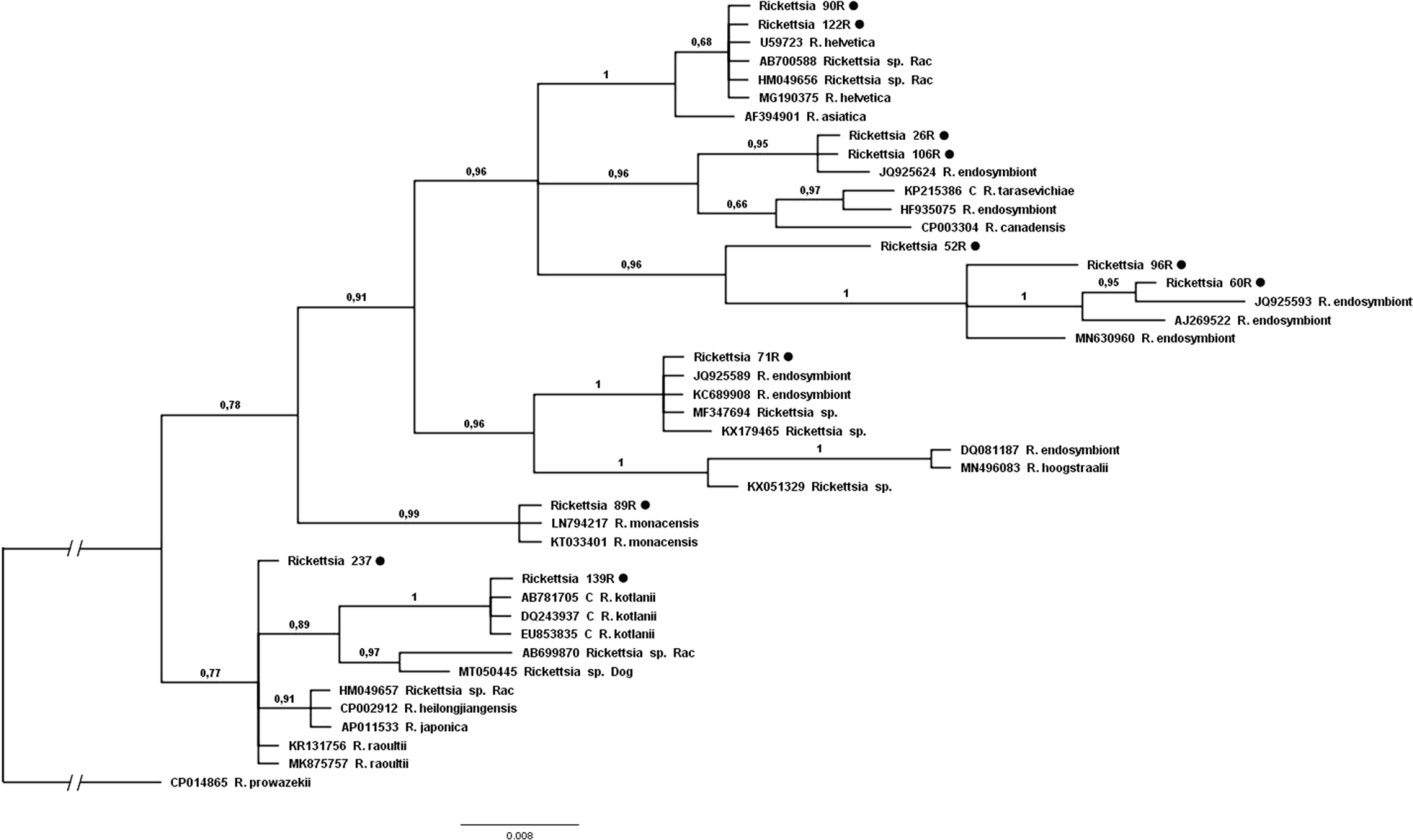
The phylogenetic relationships of the identified *Rickettsia* species. Analysis of the *gltA* partial gene based on Bayesian inference (model GTR + G + I, 2,000,000 generations). Our own isolates are marked with a* black circle*.* Rac* indicates sequences previously obtained from raccoons and available in GenBank

**Fig. 2:**
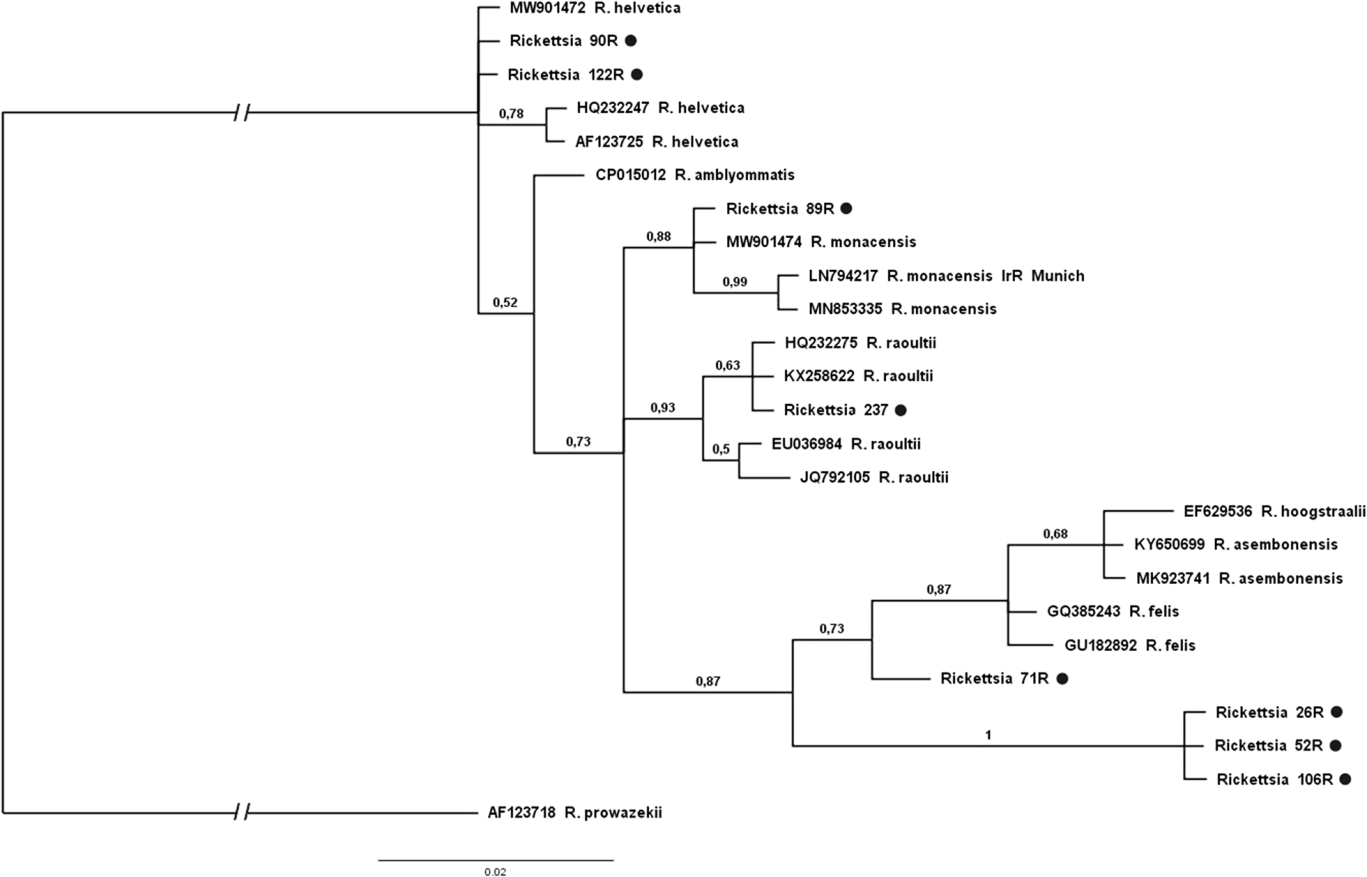
The phylogenetic relationships of the identified *Rickettsia* species. Analysis of the *ompB* partial gene based on Bayesian inference (model GTR + G + I, 1,000,000 generations). Our own isolates are marked with a* black circle*

 Using partial *gltA* gene as a marker, DNA of *Rickettsia* spp. was detected in 25 samples, i.e. 11.4% [95% confidence interval (CI) 8.7–14.7] of the tested individuals. Amplicons of the rickettsial *gltA* gene were generated for all positive PCR products, and DNA sequences obtained from 20 isolates along with homologous sequences deposited in GenBank were used for analysis. *Rickettsia helvetica* was confirmed in 13 isolates, i.e. 52% (95% CI 31.7–70.4) of positive samples. All these sequences were identical to each other and to *R. helvetica* (GenBank no. U59723). Additionally, single cases of *Rickettsia monacensis*, *Rickettsia raoultii* and *Candidatus* Rickettsia kotlanii—like (100% similarity to all three available sequences of *Candidatus* Rickettsia kotlanii for *gltA* deposited in GenBank) are reported. The remaining eight isolates [32% (95% CI 16.1–52.0) of all positive samples] showed varying degrees of similarity to different *Rickettsia* endosymbionts (Fig. 1). Out of the samples that tested positive for *gltA*, 14 amplicons of *ompB* were successfully sequenced, i.e. from *R. monacensis* (*n* = 1), *R. raoultii* (*n* = 1), *R. helvetica* (*n* = 6), and *Rickettsia* endosymbionts (*n* = 6) (Fig. 2), but only three sequences of 17 kDa were obtained, i.e. from *R. helvetica* (*n* = 2) and *Rickettsia* endosymbionts (*n* = 1).

*Rickettsia* DNA was confirmed only in skin biopsies; no isolates from the spleen or liver were positive. With the exception of evidence of *R. helvetica* in one raccoon specimen from Germany (Kassel), all samples positive for this bacterium derived from the Polish population of raccoons.

## Discussion

The role of wild animals in the life cycles of different vector-borne pathogens, and primarily those of tick-borne pathogens (TBPs), has been indicated in recent years [31, 32]. Certain species of wildlife, including wild carnivores, are suitable hosts for ticks and other haematophagous arthropods, and are also the main reservoirs of some vector-borne pathogens of medical and veterinary concern. However, for some TBPs, the role of wildlife is related to the persistence of pathogens in the environment, or is still not completely understood. On the one hand, in recent years the spectrum of TBPs affecting domestic animals and humans has increased, whilst on the other hand, due to urbanization and changes in natural ecosystems, populations of many wild species have increased, and they have adapted to environments in close proximity to human populations [33]. Therefore, investigation of the distribution of TBPs among wildlife and domestic animals is important and necessary in studies on their epidemiology and ecology [31, 33–35]. A group of wildlife of particularly interest in this context are invasive alien species, as they create new opportunities for pathogens present in the environment by increasing their abundance and their range, which may result in bidirectional pathogen transmission [36–38].

Raccoons are hosts to ticks and associated pathogens which can impact the health of humans, livestock, and indigenous wildlife. Although raccoons are widespread in Europe, only a limited number of studies on the prevalence of zoonotic agents in these animals have been undertaken to date, e.g. on *Baylisascaris procyonis* [10], *Trichinella* spp. [12], *Toxoplasma gondii* [17], *Cryptosporidium* spp. and *Enterocytozoon bieneusi* [15] and *Anaplasma phagocytophilum* [16].

Data on *Rickettsia* pathogens detected in both native and introduced raccoon populations are rather scarce. In areas endemic for *Rickettsia* pathogens in the USA, various serological investigations have shown a very high prevalence, i.e. 45.8%, of *R. rickettsii*, the causative agent of Rocky Mountain spotted fever, and 73.7% of *Rickettsia parkeri*, which is closely related to *R. rickettsii* [39, 40]. Interestingly, a very recent molecular study confirmed the presence of *Rickettsia* DNA in only a few raccoon tissue samples, i.e. out of the 39 tested raccoons from the city of New York, *Rickettsia* spp. was confirmed in three ear biopsy tissue samples and one blood sample [41]. Research carried out in Japan using molecular methods revealed low levels of *Rickettsia* infection in introduced populations of raccoons, i.e. 1.6% for *Rickettsia helvetica*, 1.5% for *Rickettsia amblyommii* and single samples for *Rickettsia felis* and *Rickettsia heliongjiangensis*. It should be noted that the tested material, i.e. blood or spleen tissue, derived from a large number of animals, i.e. 194 and 699 samples, respectively [42, 43]. There are no similar data, to the best of our knowledge, on European raccoon populations.

This study showed, to our knowledge for the first time, the presence of genetic material of members of the genus *Rickettsia* in tissue samples of free-living raccoons in Europe. The detected prevalence of *Rickettsia* spp., 11.4%, was calculated from all the tested individuals, regardless of the type of tissue examined; however, when we considered only the biopsy samples of the skin (ear) (Table 1), which was the only tissue in which the presence of *Rickettsia* DNA was confirmed, the prevalence reached 18.9% (95% CI 13.4–26.0). These results suggest that raccoons are not competent (or not yet competent) definitive hosts for *Rickettsia* spp. in Europe because *Rickettsia* infection in the present study was limited to a skin reaction. The most frequently identified rickettsiae [64% (95% CI 43.9–80.4) of all the positive samples] were members of the SFG, i.e. *R. helvetica*, *R. monacensis*, *R. raoultii* and *Candidatus* Rickettsia kotlanii-like. These results are not surprising as tick-borne rickettsiae have been reported from almost all European countries. *Ixodes ricinus* is the most widespread tick species in Europe, and is known to carry mainly *R. helvetica* and *R. monacensis*. The dominant *Rickettsia* species in Europe is *R. helvetica*, and numerous European studies have confirmed the presence of this bacterium in *I. ricinus*, although its prevalence varied greatly [44]. *Rickettsia raoultii* is the species most commonly connected with the tick* Dermacentor reticulatus*, which has undergone a rapid expansion in recent years in Europe [45, 46], and specifically in Poland [47–49].

Recording the presence of a pathogen in a vertebrate is not sufficient evidence for classifying that host species as a reservoir; it can only be classified as a candidate reservoir if its physiological and behavioural features support amplification and transmission of the pathogen to vectors, or it can be classified as a simple carrier host or a dead-end host [44]. However, it is currently considered that vertebrates can act as amplifying hosts of rickettsiae, thus contributing to their spread in ecosystems, even in the absence of systemic infection [32].

The identification of *Candidatus* Rickettsia kotlanii-like in Polish raccoons is interesting for various reasons. The genotype of *Candidatus* Rickettsia kotlanii was described in 2006 as new within the SFG in ixodid ticks from Hungary, but its potential for pathogenicity is still unknown [44]. Ours is the fourth report of this species for the entire world and the third for Europe, and specifically the first from outside Hungary. All previous reports are from studies on *Haemaphysalis* ticks, i.e. *Haemaphysalis concinna* was reported as a host for *Candidatus* Rickettsia in Europe and *Haemaphysalis megaspinosa* for *Candidatus* Rickettsia in Japan [50, 51]. Ipso facto, the identification of the DNA of *Candidatus* Rickettsia in the skin biopsy of a raccoon is the first report of this Candidatus bacterial species from a vertebrate. Additionally, our finding of *Candidatus* Rickettsia kotlanii-like may be indirect evidence of the occurrence of *H. concinna* in southwestern Poland. A recent study confirmed the presence of *H. concinna* among collections of juvenile ticks from rodent hosts and questing ticks from vegetation in western Poland. Interestingly, the occurrence of this tick in Poland has been reported only once so far, in 1953, in northwestern Pomerania, close to the border with Germany [49].

Our research on the potential reservoir role of the raccoon—a relatively new member of the carnivore fauna of Poland and the rest of Europe—in the circulation of vector-borne pathogens showed the presence of the DNA of *Rickettsia* endosymbionts in the examined tissues at a comparatively high frequency (one-third of positive isolates). We identified seven different endosymbiont strains (five of these are presented on the phylogenetic tree; Fig. 1) with varied homology to sequences previously deposited in GenBank. These endosymbionts have been previously identified as *Coxiella*, *Wolbachia*, and *Rickettsia* spp., and the relationship between them and the pathogenic bacteria which are transmitted by ticks to animals and humans remains unclear, as discussed in the literature [32]. Recent studies have revealed that rickettsial endosymbionts have negative effects on pathogenic rickettsiae within tick vectors, and that they preclude secondary infection. Other studies have addressed the positive influence of rickettsial endosymbionts on tick hosts [32]. However, the ancestral origins of these endosymbionts have yet to be elucidated, although it is speculated that they were originally animal pathogens acquired by ticks through their feeding on bacteremic hosts. These ancestral species of microorganisms probably divided into two groups: the endosymbionts, which have become specialists and completely adapted to ticks; and pathogenic bacteria, which have become generalists and are able to infect and reproduce in ticks as well as in vertebrate hosts [52]. If we accept this theory, it is understandable that reports of endosymbionts detected in vertebrate tissues are so infrequent. Therefore, in the light of our results, Noda et al. [52] appear to be justified in suggesting that there is a need to determine the potential of tick endosymbionts to emerge or reemerge as pathogens under natural conditions.

## Conclusions

To the best of our knowledge, this study provides the first evidence of the occurrence of *Rickettsia* pathogens and endosymbionts in the raccoon population of Europe. The results presented here thus indicate the need for further, broader research on different species of wild vertebrates, and ticks, as potential vectors and hosts of TBPs in natural as well as in peri-urban environments.

## Data Availability

All data generated or analysed during this study are included in the present article, and all the sequences were deposited in GenBank (accession numbers ON157065-ON157075 for *gltA* and ON157076-ON157083 for *ompB* genes).
